# Neglect and Motion Stimuli – Insights from a Touchscreen-Based Cancellation Task

**DOI:** 10.1371/journal.pone.0132025

**Published:** 2015-07-09

**Authors:** Simone Hopfner, Sonja Kesselring, Dario Cazzoli, Klemens Gutbrod, Annett Laube-Rosenpflanzer, Magdalena Chechlacz, Tobias Nef, Urs Mosimann, Stephan Bohlhalter, René M. Müri, Thomas Nyffeler

**Affiliations:** 1 Perception and Eye Movement Laboratory, Departments of Neurology and Clinical Research, Inselspital, Bern University Hospital and University of Bern, Bern, Switzerland; 2 Gerontechnology & Rehabilitation Group, University of Bern, Bern, Switzerland; 3 Nuffield Department of Clinical Neurosciences, University of Oxford, Oxford, United Kingdom; 4 Division of Cognitive and Restorative Neurology, Department of Neurology, Inselspital, Bern University Hospital and University of Bern, Bern, Switzerland; 5 Division of Computer Science, Institute for ICT-Based Management, Bern University of Applied Sciences, Biel, Switzerland; 6 Department of Experimental Psychology, University of Oxford, Oxford, United Kingdom; 7 ARTORG Center for Biomedical Engineering Research, University of Bern, Bern, Switzerland; 8 University Hospital of Old Age Psychiatry and Psychotherapy, University of Bern, Bern, Switzerland; 9 Center of Neurology and Neurorehabilitation, Luzerner Kantonsspital, Luzern, Switzerland; Eberhard Karls University of Tuebingen Medical School, GERMANY

## Abstract

**Background and Purpose:**

In stroke patients, neglect diagnostic is often performed by means of paper-pencil cancellation tasks. These tasks entail static stimuli, and provide no information concerning possible changes in the severity of neglect symptoms when patients are confronted with motion. We therefore aimed to directly contrast the cancellation behaviour of neglect patients under static and dynamic conditions. Since visual field deficits often occur in neglect patients, we analysed whether the integrity of the optic radiation would influence cancellation behaviour.

**Methods:**

Twenty-five patients with left spatial neglect after right-hemispheric stroke were tested with a touchscreen cancellation task, once when the evenly distributed targets were stationary, and once when the identic targets moved with constant speed on a random path. The integrity of the right optic radiation was analysed by means of a hodologic probabilistic approach.

**Results:**

Motion influenced the cancellation behaviour of neglect patients, and the direction of this influence (i.e., an increase or decrease of neglect severity) was modulated by the integrity of the right optic radiation. In patients with an intact optic radiation, the severity of neglect significantly decreased in the dynamic condition. Conversely, in patients with damage to the optic radiation, the severity of neglect significantly increased in the dynamic condition.

**Conclusion:**

Motion may influence neglect in stroke patients. The integrity of the optic radiation may be a predictor of whether motion increases or decreases the severity of neglect symptoms.

## Introduction

Neglect, a disabling syndrome after stroke, is defined as a deficit in detecting, responding, or orienting towards stimuli presented on the contralateral side of a brain lesion [1]. Neglect is associated with damage to an extended attentional network, which includes the parietal cortex, the superior/middle temporal cortex and the underlying insula, the ventrolateral prefrontal cortex and frontal eye field, and subcortical regions such as the caudate nucleus, the putamen, and the thalamic pulvinar [2]. Furthermore, converging evidence shows that also the disconnection of specific white matter tracts can result in neglect symptoms, such as the superior longitudinal fasciculus (SLF), the inferior longitudinal fasciculus (ILF), and the inferior fronto-occipital fasciculus (IFOF) [3].

In clinical routine, neglect diagnostic is most often performed by means of static stimuli, i.e., with paper-pencil tasks. Among them, cancellation tasks are very commonly applied, since their sensitivity to detect neglect is very high [4]. In these tasks, patients are asked to cancel targets that are evenly distributed on a sheet of paper. To assess the severity of neglect, the number of omissions as well as the starting point of cancellation are usually considered [4, 5]. For instance, whereas healthy controls begin to cancel targets on the left side [6], neglect patients with right-hemispheric brain lesions begin on the right side of the page [7]. This has been considered one of the most prominent signs of neglect [5, 8].

In everyday life, however, patients are constantly confronted with motion, i.e., moving persons or objects. More specific neuropsychological assessment tools are thus needed to test how neglect patients orient and react to moving stimuli. Previous research has consistently shown that a moving cue in the contralesional hemispace can decrease neglect severity, whereas a moving cue in the ipsilesional hemispace can increase neglect severity [9–11]. However, it is not known how visual attention in neglect patients is influenced by motion cues that are simultaneously presented in the contra- and ipsilesional hemispaces. This scenario reflects more closely the situations encountered in everyday life, where motion is rarely confined to one hemispace. Furthermore, patients with right-hemispheric stroke and left-sided neglect often have additional damage to the right optic radiation, with resulting visual field defects [12–16]. It is not known how the resulting visual field deficits influence neglect symptoms with bilateral motion cues.

In the present study, we aimed at directly comparing the cancellation behaviour of patients with left neglect after right-hemispheric stroke in a touchscreen-based cancellation task under two conditions: once when targets were stationary (static condition), and once when the same targets moved with constant speed on a random path, over the whole screen (dynamic condition). Moreover, in a control experiment, patients performed the same task in two further conditions: once when targets moved only within the left hemiscreen (i.e., dynamic left condition), and once only within the right hemiscreen (i.e., dynamic right condition). We also aimed at assessing whether damage to the visual pathways would influence cancellation behaviour under these different conditions. Since a reliable differentiation between neglect and visual field deficits is difficult to achieve with clinical confrontation testing and visual perimetry [15, 17], we evaluated whether the right optic radiation was damaged or not by means of track-wise lesion deficits analysis (www.natbrainlab.com; [18, 19]). As a comparison, a control group of age-matched healthy controls was tested with the same tasks.

We hypothesized that moving stimuli would act as an attractor of visual attention, and that the cancellation behaviour would be different in neglect patients with or without an additional damage to the optic radiation (and thus a probable left visual field defect).

## Materials and Methods

### Participants

Twenty-five patients with left-sided neglect after right-hemispheric brain damage (aged between 24 and 86, mean = 54, SD = 17.08, 9 women) and 25 healthy controls (aged between 24 and 75, mean = 54, SD = 15.75, 14 women) were included in the study. There was no significant difference with respect to age between the groups (t(23) = -.384, p = .704, 2-tailed). For patients, the mean interval between stroke onset and testing was 29 days (SD = 13.97, range 10–70 days). The patients were recruited in three different rehabilitation clinics. Neglect diagnosis was based on clinical judgement and clinical testing (Star cancellation test [20], Bells test [7], Line bisection test [20], or Complex line bisection test [21]). All participants had normal or corrected-to-normal visual acuity.

**Fig 1:**
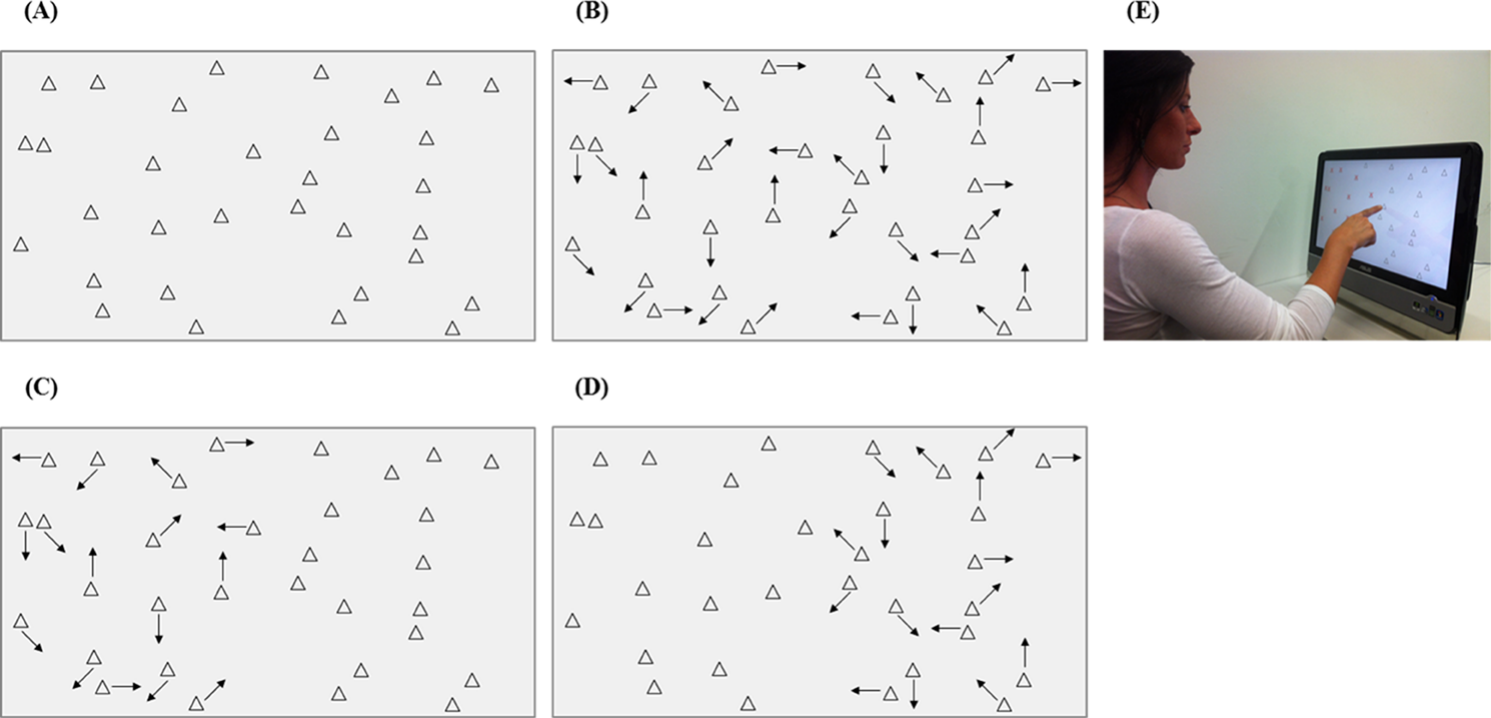
Static and dynamic conditions, and touchscreen apparatus. The four conditions performed by every participant. In the pictures, the arrows represent motion. A) Static: all targets are stationary. B) Dynamic: all targets move. C) Dynamic left: targets within the left hemiscreen move, and targets within the right hemiscreen are stationary. D) Dynamic right: targets within the right hemiscreen move, and targets within the left hemiscreen are stationary. E) Touchscreen apparatus, with a healthy participant performing the cancellation task with 32 triangular, non-coloured targets.

All participants gave written informed consent prior to the experiment. Ethical approval to conduct the study was provided by the Ethical Committee of the State of Bern. The present study was conducted in accordance with the principles of the latest version of the Declaration of Helsinki. The healthy participant (Fig 1) in this manuscript has given written informed consent (as outlined in PLOS consent form) to publish these case details. Data cannot be made publicly available due to ethical restrictions protecting patient privacy. Requests to access the data should be submitted to Prof. Thomas Nyffeler (corresponding author).

### Lesion analysis

Lesion mapping and overlap analyses of the structural MRI data were performed using MRIcron software [22], in order to identify the localization and the volume of the lesions. We applied the same procedure as Karnath et al. [23] and Karnath, Himmelbach and Rorden [24]. The used MRI sequence was dependent on the time of acquisition. If MRI was conducted within the first 48h post-stroke, then diffusion-weighted scans were used. Otherwise, T2-weighted scans were used. For every transverse slice, the boundary of the lesions was delineated directly on the individual MRI images. Both the scan and the lesion shape were then mapped into approximate MNI (Montreal Neurological Institute) space using the spatial normalization algorithm provided by SPM5 (http://www.fil.ion.ucl.ac.uk/spm/). Mapping of the lesions was performed by one of the collaborators without any knowledge of the patients’ test results. An independent, second collaborator checked the accuracy of the mapping. For some patients, only CT scans were available. In those cases, lesions were mapped directly on the T1-weighted MNI (Montreal Neurology Institute, MNI) single subject template implemented in MRIcron [25] with 1x1x1 mm resolution.

Furthermore, to evaluate the integrity of the right optic radiation, we conducted a track-wise ‘hodological’ lesion-deficit analysis [19], which is based on a recently published DTI atlas [18]. The atlas provides the probability for each voxel in the MNI space to belong to a specific white matter tract. To conduct this analysis, we used the ‘Tractotron’ software [19]. The individual pattern of the integrity of the right optic radiation was analysed by overlapping the patients’ individual lesion map with the map of the right optic radiation. The optic radiation was considered to be disconnected (binary measure) if the patients’ individual lesion map overlapped on a voxel within the optic radiation map with a probability greater than 50% (i.e., above chance level). To control for the influence of the integrity of other white matter tracts that have been shown to play an important role in neglect, we also considered the middle branch of the superior longitudinal fasciculus (SLF II; [19]), the inferior longitudinal fasciculus (ILF; [26]), and the inferior fronto-occipital fasciculus (IFOF; [27]) within the right hemisphere. These tracts were analysed according to the same procedure as for the optic radiation. We also calculated lesion volume and age to control for the influence of these variables.

### Stimulus material and apparatus

The stimulus material consisted of arrays of 32 triangular targets. The software used to present the targets was developed in cooperation with the Institute for ICT-Based Management, Division of Computer Science, Bern University of Applied Sciences, Biel, Switzerland. The software enables to design experimental conditions in which the targets are either stationary or are moving with constant speed on a random path, thus allowing to directly compare the distribution of spatial attention under static and dynamic conditions. Furthermore, thanks to an invisible and individually manipulable grid, targets can be assigned to well-defined areas of the screen. For the present study, we used a 4 x 4 grid, resulting in 16 areas. We allocated two targets to each predefined area, resulting in 32 targets in total. Since we were interested in the time course of the cancellation behaviour, a task with a low level of complexity was chosen, without any distractors or time constraint. This ensured that behavioural performance would be mainly modulated by motion (static and dynamic conditions), without potential confounds coming from distracters or time pressure. A depiction of the four conditions (static, dynamic, dynamic left, dynamic right) is shown in Fig 1a–1d.

In all dynamic conditions, all moving targets had the same constant velocity (1°/s). Targets located within the same grid area randomly changed their direction when either reaching the edge of the respective area, or when colliding with each other.

Targets were presented on a 20” touchscreen monitor (Asus ET2002T Eee Top LCD-Touchscreen-PC, 20), with a resolution of 1600 x 900 pixels, an active screen size of 25 x 44.5 cm, 32 bit colour-depth, and a refresh rate of 60 Hz (see Fig 1e). The software ensured that the exact location and time response of each target cancellation was recorded and stored.

### Experimental procedures

Participants were placed in front of the monitor, with their sagittal body-midline aligned to the midline of the computer monitor. They were instructed to touch with their right index finger all targets that they could perceive. In order to provide the participants with a visual feedback, targets were marked by a red cross once touched. Since there was no time constraint for the task, participants were instructed to verbally indicate when finished. In order to accustom patients and healthy controls to the experimental setting, a test run containing eight targets, placed on the mid-sagittal plane of the screen, was accomplished prior to the main procedure. The static and dynamic conditions were administered in counterbalanced order over participants. In patients, the experiment lasted about forty minutes, depending on patients’ individual abilities. Between conditions, patients were allowed a break of about 10 minutes. In healthy controls, the whole experiment lasted about ten minutes.

### Data analysis

The location and the time of each cancellation were assessed over four vertical columns, i.e., leftmost, left-central, right-central, and rightmost, resulting in 8 targets per column.

The detection rate in the four columns was calculated for each condition and participant, and then computed for the two groups, i.e., patients and healthy controls. Since the detection rate results evidenced a ceiling effect, the data concerning this parameter are presented descriptively and did not undergo statistical analysis. As a second parameter, the time needed until the first target was cancelled in the respective conditions and columns was analysed in the two groups. The parameter ‘time until first cancellation’ underwent a repeated-measures ANOVA with the within factors ‘condition’ (levels: static, dynamic, dynamic left, dynamic right) and ‘column’ (levels: leftmost, left-central, right-central, rightmost).

To examine whether the integrity of specific white matter pathways within the right hemisphere (i.e., optic radiation or other white matter tracts critical for neglect; SLF2, ILF, IFOF) predicted the modulation of behavioural performance in the dynamic condition compared to the static condition of the cancellation task, we performed a binary logistic regression. The modulation of behavioural performance was defined as the dependent variable (levels: improving with bilaterally presented dynamic stimuli, not improving with bilaterally presented dynamic stimuli). Based on the track-wise ‘hodological’ lesion-deficit analysis [19] described in the previous section (2.2), we defined the integrity of the respective tracts as the predictor variable (levels: intact, damaged). The same procedure was used to assess whether unilaterally presented motion in the control conditions (i.e., dynamic left, dynamic right) would significantly predict the modulation in the cancellation behaviour, each control condition compared to the static condition (levels: improving with unilaterally presented dynamic stimuli, not improving with unilaterally presented dynamic stimuli). Lesion volume and age were entered as additional predictor variables in all analyses. Each track-wise lesion deficit analysis was corrected for multiple comparisons by the Bonferroni correction. Based on the four white matter tracts analysed, the α-level was set at .0125.

Furthermore, an additional analysis was performed to follow-up the effects of the integrity of specific white matter tracts in the sub-group of neglect patients. For this analysis, the parameter ‘time until first cancellation’ underwent an additional repeated-measures ANOVA with the within factors ‘condition’ (levels: static, dynamic, dynamic left, dynamic right) and ‘column’ (levels: leftmost, left-central, right-central, rightmost), and the between factor ‘integrity of white matter tract’ (levels: intact tract, damaged tract). Post-hoc analyses were conducted by means of Bonferroni-corrected t-tests.

## Results

### Analysis of the detection rate

Healthy controls found all targets in all conditions.

Neglect patients found all targets in the left-central column, right-central column, and rightmost column in all conditions. They also found all 8 targets in the leftmost column in the dynamic left condition, whereas they showed a moderately impaired performance in the static (found targets: m = 7.84, standard error of the mean [SEM] = .945), the dynamic (m = 7.68, SEM = .198), and the dynamic right (m = 7.72, SEM = .158) conditions.

### Analysis of the time needed until the first target cancellation

#### Behavioural results (group level)

The analysis in the two groups (patients vs. healthy controls) of the time needed until the first target was cancelled revealed a significant main effect of the factor ‘column’ (F(3, 144) = 6.545, p < .001), a significant main effect of the factor ‘group’ (F(1, 48) = 5.093, p = .029), and a marginally significant main effect of the factor ‘condition’ (F(3, 144) = 2.573, p = .056). The analysis further revealed significant two-way interactions between the factors ‘condition x group’ (F(3, 144) = 3.710, p = .013), ‘column x group’ (F(3, 144) = 48.680, p < .001), and ‘condition x column’ (F(9, 432) = 4.946, p = < .001). Finally, the analysis evidenced a non-significant three-way interaction between the factors ‘condition x column x group’ (F(9, 432) = 1.283, p = .244).

**Fig 2:**
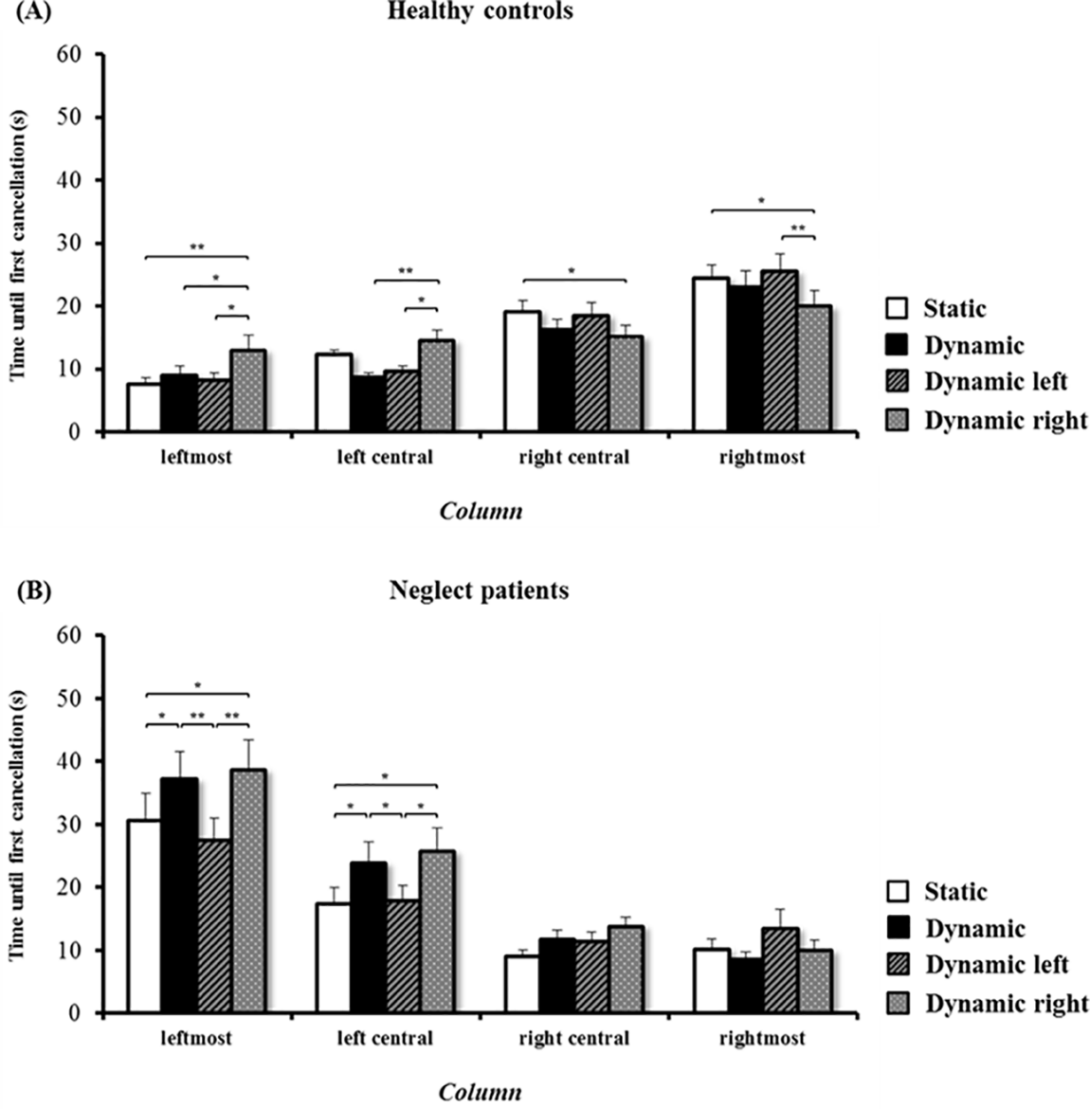
Mean time until first target cancellation in healthy controls and patients. Mean time until first cancellation in healthy controls (A) and neglect patients (B), in the four conditions (static, dynamic, dynamic left, and dynamic right), and in the respective columns: leftmost, left-central, right-central, and rightmost. Error bars represent the standard error of the mean (SEM). Asterisks depict significant post-hoc tests (* p < .05).

In a further analysis, the time needed until the first target was cancelled was analysed in patients and healthy controls separately. In healthy controls, the analysis of the time needed until the first target was cancelled revealed no significant effect of the factor ‘condition’ (F(3, 72) = 1.138, p = .340). Irrespective of the column, healthy controls needed equivalent time until the first cancellation in all conditions. The analysis further revealed a significant effect of the factor ‘column’ (F(3, 72) = 18.343, p < .001). Irrespective of the condition, healthy controls began their search in the left part of the screen, as reflected by the shorter times until the first target was cancelled in the leftmost and the left-central column as compared to the times in the right-central and the rightmost column. Finally, the analysis revealed a significant interaction ‘condition x column’ (F(9, 216) = 3.82, p < .001). The results concerning this interaction and the corresponding post-hoc tests are depicted in Fig 2a. In the dynamic right condition, healthy controls started their search significantly later in the leftmost and the left-central column, and significantly earlier in the right-central and the rightmost column, as compared to the other conditions.

The analysis of the time needed until the first target was cancelled in neglect patients revealed a significant effect of the factor ‘condition’ (F(3, 72) = 3.706, p = .015). Irrespective of the column, neglect patients began their search earlier in the conditions static and dynamic left than in the conditions dynamic and dynamic right. The analysis further revealed a significant effect of the factor ‘column’ (F(3, 72) = 33.355, p < .001). Irrespective of the condition, neglect patients started their search later in the leftmost column than in all other three columns. Finally, the analysis revealed a significant interaction ‘condition x column’ (F(9, 216) = 2.779, p = .004). The results concerning this interaction and the corresponding post-hoc tests are depicted in Fig 2b. Neglect patients started their search in the left hemiscreen (leftmost and left-central column) significantly earlier in the conditions static and dynamic left than in the conditions dynamic and dynamic right. Conversely, there were no significant differences for the right hemiscreen (right-central and rightmost columns).

#### Results analysed according to the integrity of the white matter tracts (subgroup level)

A binary logistic regression was conducted to analyse whether the integrity of specific white matter tracts within the right hemisphere would significantly predict a performance change in the dynamic condition with respect to the static one, when controlling for lesion volume and age. The analysis revealed that the integrity of the right optic radiation was a significant predictor of changes in performance with dynamic (i.e., bilaterally presented motion) compared to static stimuli (β = 3.639, p = .011), after controlling for lesion volume and age. The integrity of the other analysed white matter tracts within the right hemisphere did not significantly predict changes in performance with dynamic compared to static stimuli: SLF 2 (β = .233, p = .845), ILF (β = -1.462, p = .265), IFOF (β = -20.936, p > .99); again, after controlling for lesion volume and age.

The same analysis was conducted for the two control conditions, where motion was presented unilaterally (i.e., dynamic left and dynamic right condition). This analysis revealed that the integrity of the right optic radiation was a significant predictor of changes in performance in the dynamic left compared to the static condition (β = 5.297, p = .007), but not in the dynamic right compared to the static condition (β = .900, p = .382), after controlling for lesion volume and age. No other analysed white matter tract within the right hemisphere significantly predicted changes in performance with unilateral left- or right-sided motion: SLF 2 (dynamic left: β = -.605, p = .546; dynamic right: β = .749, p = .540); ILF (dynamic left: β = -1.723, p = .179; dynamic right: β = .776, p = .551); IFOF (dynamic left: β = -22.116, p < .99; dynamic right: β = 20.797, p < .99); again, after controlling for lesion volume and age.

**Fig 3:**
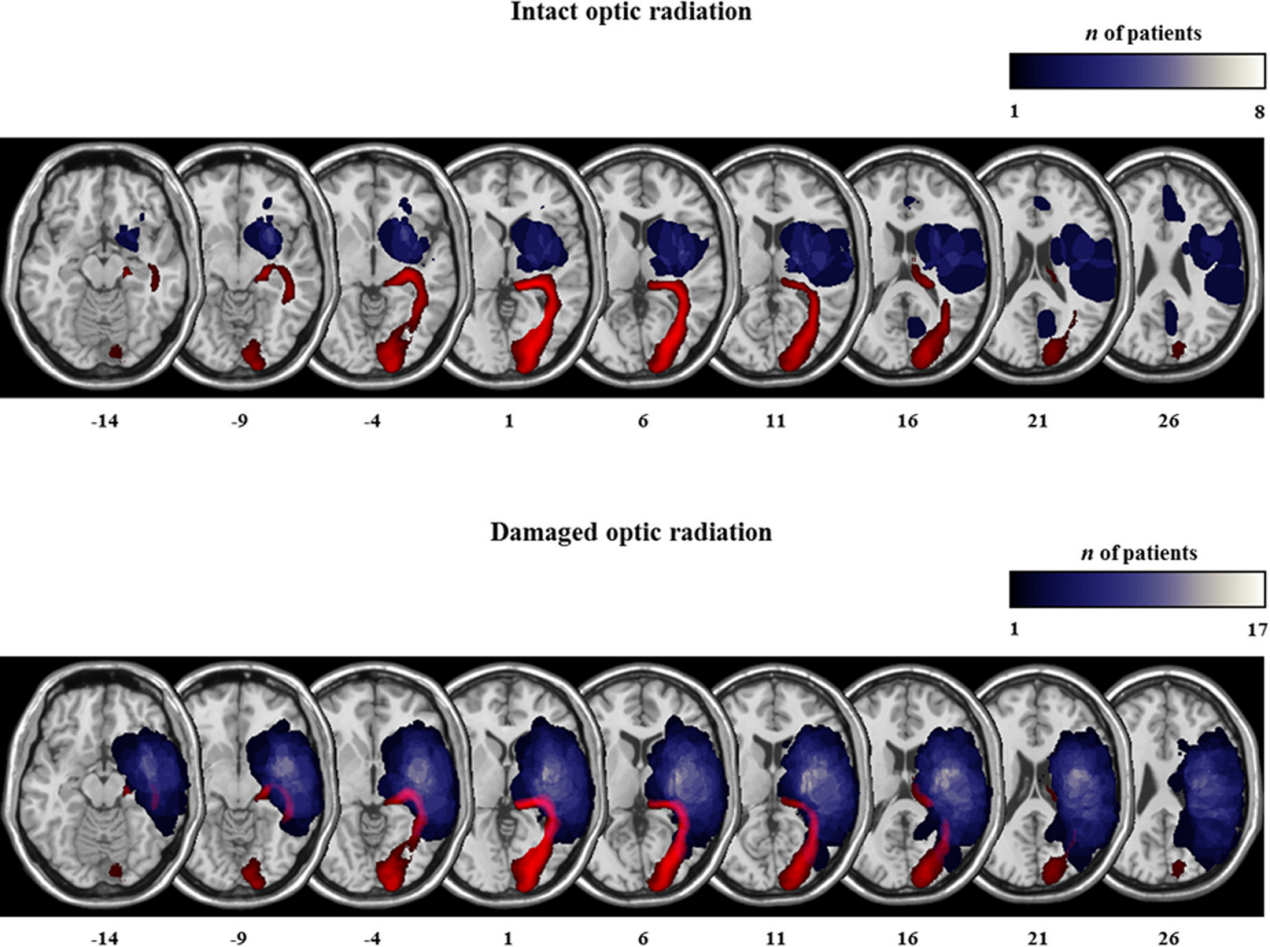
Lesion mapping. Lesion location and overlap map for neglect patients with intact and damaged right optic radiation. Lesion overlap plotted onto the ch2.nii.gz template of the MNI brain. Lesions are plotted on axial slices oriented according to neurological convention. The slices of the brain are depicted in 5mm ascending steps. The Z position of each axial slice in the MNI Talairach stereotaxic space is presented at the bottom of the figures. The number of patients with damage involving a specific region is colour-coded according to the legend. The optic radiation is based on a recently published DTI atlas (18), and is represented in red colour.

Furthermore, a separate binary logistic regression revealed that lesion volume per se could not predict the observed change in performance, neither in the dynamic condition (β = -.010, p = .191), nor in the dynamic left condition (β = .006, p = .354). Fig 3 shows the localisation and the degree of overlap of the brain lesions, transferred to standard atlases.

Concerning the dynamic condition, omnibus test of the model coefficients regarding the integrity of the right optic radiation revealed that the model was valid (p = .008). Furthermore, the model correctly classified 87.5% of patients with intact optic radiation, showing a performance increase in the dynamic condition compared to the static condition. On the other hand, 82.3% of patients with damaged optic radiation were classified correctly by the model, showing that the performance either remained stable or decreased in the dynamic condition compared with the static condition. Furthermore, omnibus test of the model coefficients for the two control conditions revealed that the model was only valid for the dynamic left condition, but not for the dynamic right condition (p = .002 and p = .783, respectively). For the dynamic left condition, the model correctly classified 87.5% of patients with intact optic radiation, showing a performance increase in the dynamic left condition compared to the static condition. On the other hand, 64.7% of patients with damaged optic radiation were correctly classified by the model, showing that the performance either remained stable or decreased in the dynamic left condition compared with the static condition.

**Fig 4:**
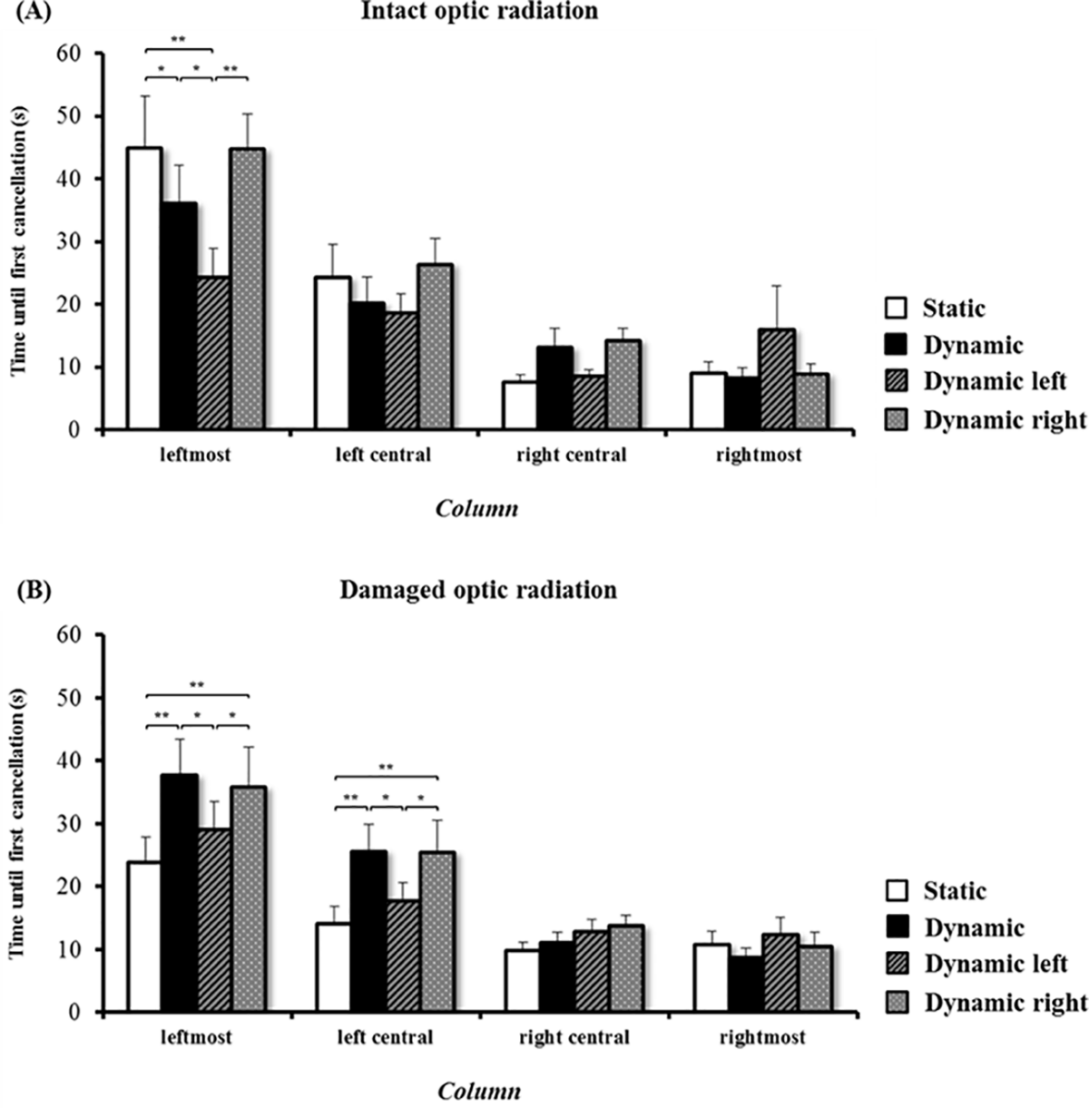
Analysis in sub-groups. Mean time until first cancellation in neglect patients with intact optic radiation (A) and damaged optic radiation (B), in the four conditions (static, dynamic, dynamic left, and dynamic right), and in the respective columns: leftmost, left-central, right-central, and rightmost. Error bars represent the standard error of the mean (SEM). Asterisks depict significant post-hoc tests (* p < .05; ** p < .01).

Based on these results, sub-groups were created according to the integrity of the right optic radiation (i.e., patients with intact (n = 8) or damaged (n = 17) optic radiation), and the analysis of the performance was re-run with this additional factor. The analysis of the time needed until the first target was cancelled in the two sub-groups of patients revealed a significant main effect of the factor ‘condition’ (F(3, 69) = 2.973, p = .038), a significant main effect of the factor ‘column’ (F(3, 69) = 31.482, p < .001), and a significant interaction ‘condition x column’ (F(9, 207) = 3.131, p = .001). There was no significant effect of the factor ‘optic radiation integrity’ (F(1, 23) = .205, p = .655), or of the interactions ‘optic radiation integrity x condition’ (F(3, 69) = 2.089, p = .110) and ‘optic radiation integrity x column’ (F(3, 69) = .584, p = .627). However, there was a highly significant three-way interaction between the factors ‘condition x column x optic radiation integrity’ (F(9, 207) = 2.847, p = .003). The results concerning this interaction and the corresponding post-hoc tests are depicted in Fig 4. Neglect patients with an intact optic radiation (Fig 4a) started their search in the leftmost column significantly earlier in the dynamic condition and the dynamic left condition than in the static condition, i.e., their performance improved with motion. In the dynamic right condition, no significant difference was found as compared to the static condition. Differently, neglect patients with a damaged optic radiation (Fig 4b) started their search in the leftmost column significantly later in the dynamic condition and the dynamic right condition than in the static condition, i.e., their performance worsened with motion. This pattern was similar in the left-central column.

## Discussion

In the present study, a group of patients suffering from left-sided neglect after a right-hemispheric stroke and an aged-matched group of healthy controls performed a cancellation task on a touchscreen monitor, under static and dynamic conditions.

In the static condition, patients showed typical neglect behaviour. They began their search on the right side of the screen and required more time until the first cancellation of a left-sided target [4, 5, 8, 28]. In contrast, healthy controls cancelled targets from the left to the right. The ceiling effect observed in the detection rate in our patients probably reflects the relatively low level of complexity of the task, since no distractors and no time constraint were used. However, the primary aim of the present study was to analyse the time course of the cancellation behaviour under static and dynamic conditions. As shown by our results, the analysis of the time until the first cancellation across the four columns was a sufficiently sensitive parameter to capture a typical neglect cancellation pattern [8].

In the dynamic condition, neglect severity increased on a group level, i.e., patients required significantly more time until the first cancellation of a left-sided target with moving stimuli. However, a sub-analysis based on track-wise lesion deficits analysis showed that the behavioural pattern in the cancellation task significantly differed between patients with and without damage to the right optic radiation. In neglect patients with an intact optic radiation, the cancellation behaviour significantly improved in the dynamic condition as compared with the static one. The time needed until the first cancellation of a target on the left part of the screen was significantly shorter in the dynamic condition than in the static one. This is in line with previous research, showing that moving cues on the contralesional side can attract visual attention towards the impaired hemifield and in turn decrease neglect severity [9, 29]. In neglect patients with a damaged optic radiation, the dynamic condition triggered a significant increase in neglect severity. The time required until the first cancellation of a target on the left side of the screen was significantly longer in the dynamic condition than in the static one. This suggests that the presence of an additional lesion of the right optic radiation may have a critical influence on cancellation behaviour in neglect patients. The probable visual field defect—resulting from the optic radiation damage within the right hemisphere—may have prevented a left-sided cueing effect, letting in turn right-sided moving targets fully attract visual attention, and thereby increasing the severity of neglect [30, 31].

Such an interpretation is also supported by the results of our control experiment, where the influence of unilaterally left- or right-sided motion was evaluated. Unilateral left-sided motion (i.e., dynamic left condition) resulted in an improvement of cancellation behaviour in patients with an intact optic radiation, but not in patients with damage to the optic radiation. This finding is interesting in the context of previous research that has consistently shown how a moving cue in the contralesional hemispace can decrease neglect severity [9, 29]. Unfortunately, in these studies no detailed information about the integrity of the optic radiation and possible visual field defects was given.

Again in line with the literature [10, 31], we found that unilateral right-sided motion worsened the performance in patients with a damaged optic radiation. Interestingly, a significant change in performance in the dynamic right condition compared to the static condition was also found in healthy controls. Healthy controls show a ‘left to right’ cancellation behaviour in both conditions. However, in the dynamic right condition, their cancellation starts significantly earlier on the right side of the screen and significantly later on the left side of the screen, i.e., cancellation performance is biased towards the side of motion. This underlines the important role of motion, attracting visual attention, and thus resulting in an attentional shift towards the moving stimuli [32].

Although it remains unclear whether damage of the right optic radiation causatively modulates cancellation behaviour, it is interesting to note that we found a robust association. For the dynamic condition, omnibus test of the model coefficients revealed that our model is valid. 87.5% of patients with intact optic radiation and 82.3% of patients with damaged optic radiation were classified correctly. Lesion volume per se or lesions of other white matter tracts critical for neglect (SLFII, ILF, IFOF), however, did not significantly predict the behavioural modulation in the dynamic conditions. In addition, it is unlikely that the increase in neglect severity with motion can be solely explained by visual field deficits, rather than by their interaction with visual attention. First, targets moved very slowly (i.e., 1°/sec) within a predefined area. Second, and more important, cancellation behaviour did not significantly deteriorate in the right side of the screen in the dynamic and dynamic right condition, as one would expect if a visual field deficit per se would make visual pursuit more difficult.

Visual orienting towards a moving stimulus, i.e., orienting the eyes and the head [32], has been often attributed to the midbrain superior colliculus [33–36]. It has also been proposed that the superior colliculus could play a major role in unconsciously orienting attention towards motion in neglect patients [9, 29]. Our findings extend this view, suggesting that lower visual areas relying on the geniculo-striate pathway [15] might also play a critical role in orienting visual attention towards moving stimuli in neglect patients.

The question whether neglect severity in patients is modulated by motion may also be clinically relevant. The results presented in this study, showing that the integrity of the right optic radiation critically influences the cancellation behaviour under static and dynamic conditions may have potential therapeutic value.

Our study has some limitations. We used a probabilistic approach using track-wise lesion deficits analysis. Diffusion tensor imaging (DTI) sequences would certainly have been more reliable for an exact analysis of the involved fibre tracts. Nevertheless, the results of our study may represent a first step and help clinicians, since they suggests that an intact or damaged optic radiation may be a predictor of whether motion increase or decrease neglect severity in a particular patient. A detailed assessment of the MR images, specifically looking for damage of the optic radiation, may thus be of value.

## References

[1] Heilman KM, Watson RT, Valenstein E. Neglect and related disorders In: Heilman KM, Valenstein E, editors. Clinical neuropsychology. 4 ed. London, UK: Oxford University Press; 2003 pp. 296–346.

[2] Karnath HO, Rorden C. The anatomy of spatial neglect. Neuropsychologia. 2012;50(6): 1010–1017. 10.1016/j.neuropsychologia.2011.06.027 WOS:000304724500002. 21756924 PMC3348466

[3] Doricchi F, Thiebaut de Schotten M, Tomaiuolo F, Bartolomeo P. White matter (dis)connections and gray matter (dys)functions in visual neglect: gaining insights into the brain networks of spatial awareness. Cortex. 2008;44(8): 983–995. Epub 2008/07/08. 10.1016/j.cortex.2008.03.006 . 18603235

[4] Ferber S, Karnath HO. How to assess spatial neglect—Line bisection or cancellation tasks? Journal of Clinical and Experimental Neuropsychology. 2001;23(5): 599–607. 10.1076/jcen.23.5.599.1243 WOS:000171476200004. 11778637

[5] Azouvi P, Samuel C, Louis-Dreyfus A, Bernati T, Bartolomeo P, Beis JM, et al Sensitivity of clinical and behavioural tests of spatial neglect after right hemisphere stroke. J Neurol Neurosurg Psychiatry. 2002;73(2): 160–166. Epub 2002/07/18. 12122175 10.1136/jnnp.73.2.160 PMC1737990

[6] Rousseaux M, Beis JM, Pradat-Diehl P, Martin Y, Bartolomeo P, Bernati T, et al Presenting a battery for assessing spatial neglect. Norms and effects of age, educational level, sex, hand and laterality. Rev Neurol (Paris). 157: 1385–1400. 11924007

[7] Gauthier L, Dehaut F, Joanette Y. The Bells test: A quantitative and qualitative test for visual neglect. International Journal of Clinical Neuropsychology. 1989;11: 49–54.

[8] Vuilleumier P. Hemispatial neglect In: Godefroy O, Bogousslavsky J, editors. The Behavioral and Cognitive Neurology of Stroke. New York: Cambridge University Press; 2007 pp. 148–197.

[9] Butter CM, Kirsch NL, Reeves G. The effect of lateralized dynamic stimuli on unilateral spatial neglect following right-hemisphere lesions. Restorative Neurology and Neuroscience. 1990;2(1): 39–46. WOS:A1990EV45600005. 10.3233/RNN-1990-2105 21551871

[10] Mattingley JB, Bradshaw JL, Bradshaw JA. Horizontal visual-motion modulates focal attention in left unilateral spatial neglect. Journal of Neurology Neurosurgery and Psychiatry. 1994;57(10): 1228–1235. 10.1136/jnnp.57.10.1228 WOS:A1994PN02600015. PMC485492 7931385

[11] Plummer P, Dunai J, Morris ME. Understanding the effects of moving visual stimuli on unilateral neglect following stroke. Brain and Cognition. 2006;60(2):156–165. 10.1016/j.bandc.2005.11.001. 16466838

[12] Cassidy TP, Bruce DW, Lewis S, Gray CS. The association of visual field deficits and visuo-spatial neglect in acute right-hemisphere stroke patients. Age and Ageing. 1999;28(3): 257–60. 10475860 10.1093/ageing/28.3.257

[13] Halligan PW. Hemianopia and visual neglect: a question of balance? Journal of Neurology, Neurosurgery & Psychiatry. 1999;67(5): 565–566. 10.1136/jnnp.67.5.565 PMC1736650 10519856

[14] Halligan PW, Marshall JC, Wade DT. Do visual field deficits exacerbate visuo-spatial neglect? Journal of Neurology, Neurosurgery & Psychiatry. 1990;53(6): 487–491. 10.1136/jnnp.53.6.487 PMC1014208 2380729

[15] Muller-Oehring EM, Kasten E, Poggel DA, Schulte T, Strasburger H, Sabel BA. Neglect and hemianopia superimposed. Journal of Clinical and Experimental Neuropsychology. 2003;25(8): 1154–1168.: 10.1076/jcen.25.8.1154.16727 WOS:000186631900010. 14566587

[16] Vallar G, Perani D. The anatomy of unilateral neglect after right-hemisphere stroke lesions. A. Neuropsychologia. 1986;24(5): 609–622. Epub 1986/01/01. . 3785649 10.1016/0028-3932(86)90001-1

[17] Kerkhoff G, Schindler I. Hemi-neglect versus hemianopia. Differential diagnosis. Fortschr Neurol Psychiatr. 1997;65(6): 278–289. 10.1055/s-2007-996332 . 9273345

[18] Thiebaut de Schotten M, Ffytche DH, Bizzi A, Dell'Acqua F, Allin M, Walshe M, et al Atlasing location, asymmetry and inter-subject variability of white matter tracts in the human brain with MR diffusion tractography. Neuroimage. 2011;54(1): 49–59. Epub 2010/08/05. 10.1016/j.neuroimage.2010.07.055 . 20682348

[19] Thiebaut de Schotten M, Tomaiuolo F, Aiello M, Merola S, Silvetti M, Lecce F, et al Damage to white matter pathways in subacute and chronic spatial neglect: a group study and 2 single-case studies with complete virtual "in vivo" tractography dissection. Cereb Cortex. 2014;24(3): 691–706. Epub 2012/11/20.: 10.1093/cercor/bhs351 . 23162045

[20] Wilson B, Cockburn J, Halligan PW. The Behavioural Inattention Test. Bury St. Edmunds, UK: Thames Valley Test Company; 1987.

[21] Butter CM, Mark VW, Heilman KM. An experimental analysis of factors underlying neglect in line bisection. J Neurol Neurosurg Psychiatry. 1988;51(12): 1581–1583. Epub 1988/12/01. ; PubMed Central PMCID: PMCPmc1032779. 3221227 10.1136/jnnp.51.12.1581 PMC1032779

[22] Rorden C, Karnath HO, Bonilha L. Improving lesion-symptom mapping. J Cogn Neurosci. 2007;19(7): 1081–1088. Epub 2007/06/23. 10.1162/jocn.2007.19.7.1081 . 17583985

[23] Karnath HO, Berger MF, Kuker W, Rorden C. The anatomy of spatial neglect based on voxelwise statistical analysis: A study of 140 patients. Cerebral Cortex. 2004;14(10): 1164–1172. 10.1093/cercor/bhh076 WOS:000223895000011. 15142954

[24] Karnath HO, Himmelbach M, Rorden C. The subcortical anatomy of human spatial neglect: putamen, caudate nucleus and pulvinar. Brain. 2002;125: 350–360. 10.1093/brain/awf032 WOS:000173755900013. 11844735

[25] Rorden C, Brett M. Stereotaxic display of brain lesions. Behav Neurol. 2000;12(4): 191–200. Epub 2001/09/25. . 11568431 10.1155/2000/421719

[26] Bird CM, Malhotra P, Parton A, Coulthard E, Rushworth MF, Husain M. Visual neglect after right posterior cerebral artery infarction. J Neurol Neurosurg Psychiatry. 2006;77(9): 1008–1012. Epub 2006/06/15. 10.1136/jnnp.2006.094417 ; PubMed Central PMCID: PMCPmc2077751. 16772354 PMC2077751

[27] Urbanski M, Thiebaut de Schotten M, Rodrigo S, Catani M, Oppenheim C, Touze E, et al Brain networks of spatial awareness: evidence from diffusion tensor imaging tractography. J Neurol Neurosurg Psychiatry. 2008;79(5): 598–601. Epub 2007/11/10. 10.1136/jnnp.2007.126276 ; PubMed Central PMCID: PMCPmc2386830. 17991702 PMC2386830

[28] Heilman KM, Watson RT, Valenstein E. Spatial neglect In: Karnath H-O, Milner DA, Vallar G, editors. The Cognitive and Neural Bases of Spatial Neglect. New York: Oxford University Press; 2002 pp. 416.

[29] Butter CM, Kirsch N. Effect of lateralized kinetic visual cues on visual search in patients with unilateral spatial neglect. Journal of Clinical and Experimental Neuropsychology. 1995;17(6): 856–867. 10.1080/01688639508402435 WOS:A1995TM77600006. 8847392

[30] Mattingley JB, Pierson JM, Bradshaw JL, Phillips JG, Bradshaw JA. To see or not to see: the effects of visible and invisible cues on line bisection judgements in unilateral neglect. Neuropsychologia. 1993;31(11): 1201–1215. Epub 1993/11/01. . 8107981 10.1016/0028-3932(93)90068-b

[31] Riddoch MJ, Humphreys GW. The effect of cueing on unilateral neglect. Neuropsychologia. 1983;21(6): 589–599. Epub 1983/01/01. . 6664478 10.1016/0028-3932(83)90056-8

[32] Wolfe JM, Horowitz TS. What attributes guide the deployment of visual attention and how do they do it? Nat Rev Neurosci. 2004;5(6): 495–501 15152199 10.1038/nrn1411

[33] Cynader M, Berman N. Receptive-field organization of monkey superior colliculus. J Neurophysiol. 1972;35(2): 187–201. Epub 1972/03/01. . 4623918 10.1152/jn.1972.35.2.187

[34] Schiller PH, Koerner F. Discharge characteristics of single units in superior colliculus of the alert rhesus monkey. J Neurophysiol. 1971;34(5): 920–936. Epub 1971/09/01. . 4999593 10.1152/jn.1971.34.5.920

[35] Krauzlis RJ, Lovejoy LP, Zenon A. Superior colliculus and visual spatial attention. Annu Rev Neurosci. 2013;36: 165–182. Epub 2013/05/21. 10.1146/annurev-neuro-062012-170249 . 23682659 PMC3820016

[36] Lovejoy LP, Krauzlis RJ. Inactivation of primate superior colliculus impairs covert selection of signals for perceptual judgments. Nat Neurosci. 2010;13(2): 261–266. Epub 2009/12/22. 10.1038/nn.2470 ; PubMed Central PMCID: PMCPmc3412590. 20023651 PMC3412590

